# Controversy and consensus on a clinical pharmacist in primary care in the Netherlands

**DOI:** 10.1007/s11096-016-0360-z

**Published:** 2016-07-29

**Authors:** Ankie C. M. Hazen, Aletta W. van der Wal, Vivianne M. Sloeserwij, Dorien L. M. Zwart, Johan J. de Gier, Niek J. de Wit, Anne J. Leendertse, Marcel L. Bouvy, Antoinette A. de Bont

**Affiliations:** 1 Department of General Practice, Julius Center for Health Sciences and Primary Care, University Medical Center Utrecht, Utrecht, The Netherlands; 2 Department of Pharmacotherapy and Pharmaceutical Care, University of Groningen, Groningen, The Netherlands; 3 Division of Pharmacoepidemiology and Clinical Pharmacology, Department of Pharmaceutical Sciences, Utrecht University, Utrecht, The Netherlands; 4 Institute for Policy and Management in Health Care, Erasmus University Rotterdam, Rotterdam, The Netherlands

**Keywords:** Clinical pharmacist, General practitioner, Integrated care, Netherlands, Primary care, Q method

## Abstract

*Background* Controversy about the introduction of a non-dispensing pharmacist in primary care practice hampers implementation. *Objective* The aim of this study is to systematically map the debate on this new role for pharmacists amongst all stakeholders to uncover and understand the controversy and consensus. Setting: Primary health care in the Netherlands. *Method* Q methodology. 163 participants rank-ordered statements on issues concerning the integration of a non-dispensing pharmacist in primary care practice. Main outcome measure: Stakeholder perspectives on the role of the non-dispensing pharmacist and pharmaceutical care in primary care. *Results* This study identified the consensus on various features of the non-dispensing pharmacist role as well as the financial, organisational and collaborative aspects of integrating a non-dispensing pharmacist in primary care practice. Q factor analysis revealed four perspectives: “the independent community pharmacist”, “the independent clinical pharmacist”, “the dependent clinical pharmacist” and “the medication therapy management specialist”. These four perspectives show controversies to do with the level of professional independency of the non-dispensing pharmacist and the level of innovation of task performance. *Conclusion* Despite the fact that introducing new professional roles in healthcare can lead to controversy, the results of this Q study show the potential of a non-dispensing pharmacist as a pharmaceutical care provider and the willingness for interprofessional collaboration. The results from the POINT intervention study in the Netherlands will be an important next step in resolving current controversies.

## Impact on practice

- Most primary care professionals recognize the need for more integration of pharmaceutical care into daily primary care practice.
- General practitioners and community pharmacists regard the introduction of the non-dispensing pharmacist as a possible route to integrate pharmaceutical care into practice.
- Most primary care professionals agree that the non-dispensing pharmacist should be an integral part of the primary care team, offering consultations to vulnerable patients with polypharmacy.
- Although further separation of pharmaceutical care and drug dispensing is considered as the key paradigm shift, there is discussion about the best way to implement this.

## Introduction

Co-locating a non-dispensing pharmacist (NDP) in primary care practice, including shared use of patients’ medical records, is expected to improve interprofessional collaboration and communication and thus effective patient-centred medication management services [1]. However, controversy about this new role for pharmacists is hampering implementation. Different perceptions have led to significant barriers preventing pharmacists from expanding their roles as pharmaceutical care providers. The barriers include lack of mandate, legitimacy, effectiveness and readiness to embrace change [2]. Currently, NDPs have been integrated successfully in primary care practice in only a limited number of health care settings, mainly in Great Britain, the United States, and Canada [3–5].

Interprofessionality is an essential feature of healthcare development [6], reflected in the willingness to work in interprofessional teams [7]. Yet, introducing new roles in healthcare practices puts professional boundaries under pressure [8]. New roles lead to the substitution of labour, including reallocation of resources and control. Consequently, it has an impact on dominance and authority, fed by the implicit wish to maintain established arrangements for healthcare delivery and by scepticism about the feasibility and effectiveness of related professionals working jointly [6].

Despite the identified positive attitude to team-based work, attempts to introduce the NDP to primary care practice have led to debate, as evidenced by several qualitative studies on stakeholder experiences with NDPs in primary care practices [9, 10].

## Aim of the study

In this Q-study we systematically map the debate on the introduction of NDPs in primary care practice amongst all involved stakeholders to uncover and understand the controversy and consensus.

## Ethics approval

This Q study is part of the POINT project, which aims to evaluate the effect of integration of an NDP in general practice with regard to the quality and safety of pharmacotherapy [11]. This project is exempted of formal medical-ethical approval by the Medical Ethical Committee University Medical Centre Utrecht (METC protocol number 13-432C).

## Method

### Research design

Q methodology [12] was used to disclose different viewpoints on the value and position of the NDP in primary healthcare. The Q method is a robust and hybrid qualitative–quantitative technique that provides a basis for the systematic study of subjectivity and accentuates shared understanding [13]. A Q study consists of three steps: construction of the Q set, performing Q sorting and analysis of obtained data [14, 15].

*Step 1* Constructing the Q set.

The first step is the collection of statements broadly covering the debate on the subject at hand. In Q methodological terms this is called “the concourse” [14, 15]. The concourse on integration of an NDP in primary care was based on the literature and collected from six interviews with pharmaceutical and medical experts. From this concourse we drew a subset of 116 statements. Since careful consideration of the context is helpful to a better understanding of the debate on NDP integration, we deliberately added a number of general statements on improving pharmaceutical care in primary care. The subset of 116 statements was stripped of double and comparable statements and condensed to a Q set of 37 statements (Table 1). The statements were evaluated by a group of experts who were both pharmacists, general practitioners (GP) and researchers with experience in Q methodological studies. They refined the statement set to improve readability and clarity. Next, statements were assessed and sorted by a small group of general practitioners and pharmacists. Finally, statements were again refined and improved. The result was the final Q set which was considered representative for the issues raised on integrating an NDP in primary care. Quoted statements were originally phrased in Dutch.

**Table 1:** Q set of 37 statements and idealized Q sort for the four factors representing perceptions of integration of an NDP in primary care clinics

Statements		Factor A: independent clinical pharmacist	Factor B: independent community pharmacist	Factor C: dependent clinical pharmacist	Factor D: medication therapy management specialist
1.	With the introduction of the NDP confusion arises about whom the patient can ask questions related to medication	−1*	0	0*	0
2.	The GP wishes to minimize the number of other healthcare providers in general practice	−1	0*	−2*	−1
3.	**The NDP poses a risk to patient safety due to the resulting formation of an additional link between prescription and delivery**	−**2**	−**1**	−**3**	−**2**
4.	The patient has more confidence in the NDP than in the community pharmacist	0	−1*	1	1
5.	The community pharmacist is insufficiently informed about the pharmacotherapy of the individual patient	1*	−2*	0	−1
6.	**The NDP improves adherence**	**2**	**1**	**2**	**1**
7.	A community pharmacists’ primary concern includes the financial status of the pharmacy business	−2	−1	0*	−2
8.	**The health insurance company pays too little for pharmaceutical care**	**2**	2*	**1**	**1**
9.	A fee for practice costs for community pharmacists is essential to enable delivery of pharmaceutical care	1	2*	1	−1*
10.	**Earmarked funding for pharmaceutical care in general practice should be initiated**	**1**	**1**	**2**	**1**
11.	The NDP loses its independent position as healthcare provider as an employee of a general practice	−2	0	−1	0
12.	GP care will be unnecessarily expensive by nationwide introduction of the NDP	−2*	0	0	−1
13.	The GP has insufficient knowledge of medication	1	1	−1*	2
14.	*The tasks of the NDP and the community pharmacist are different*	1	0*	1	1
15.	The knowledge of the NDP about clinical pharmacology is essential in general practice	3*	1	1	1
16.	**Shared training in the GP’s and pharmacist’s educational programmes improves pharmaceutical care**	**1**	**2**	**3**	**1**
17.	To improve pharmaceutical care, the community pharmacist needs to give advice about the choice of medication	0	2*	0	0
18.	**The added value of the NDP is the care of the individual patient**	**1**	**1**	**2**	**3**
19.	Medication reviews should take place in general practice	0	−2*	1*	0
20.	**Access to medical records is an essential prerequisite for pharmaceutical care**	**3**	3*	**2**	**2**
21.	**The GP and NDP share a common goal in the pharmacotherapy of the patient**	**2**	**3**	**3**	**2**
22.	Information on medication provided to the patient by the community pharmacist does not sufficiently reflect the GP’s advices	0*	−1	0	2*
23.	The inferior position of the pharmacist relative to the GP impedes medication safety	0	0*	−2*	−1
24.	*The NDP will take on the fun part of the community pharmacist’s work*	−1*	0	0	0
25.	Without proactive identification of patients with potential drug therapy problems, the NDP has no added value	0	1*	−1	3*
26.	The advice on pharmacotherapy and the dispensing of the medication should be separated	0	−2*	0*	0
27.	The community pharmacist is not skilled to perform a patient consultation	0	−3*	0	0
28.	To enable a successful collaboration it is necessary that GP and NDP are working in the same organisation	2*	0	0	0
29.	**The NDP is doing work that can be done more adequately by a practice nurse**	−**3**	−2*	−**3**	−**3**
30.	*The logistics in the community pharmacy can be coordinated more adequately by someone with a bachelor’s degree*	0	0	0	0
31.	The education of the patient about their medication use should be linked to the dispensing of the medication	−1	1*	−1	−2*
32.	Pharmaceutical care (including the dispensing of the medication) can best be accommodated at a general practice	0	−3*	−1	−1
33.	*The community pharmacist should focus solely on counselling on pharmacotherapy*	−1	−1	−1	0
34.	The NDP must be an independent prescriber	0*	0*	−2	−3
35.	*A general practice with 10,000 patients is too small to employ a full*-*time NDP*	−1*	0	−1*	0
36.	The NDP cannot be employed at a community pharmacy due to conflict of interest	−1	−1*	1*	−1
37.	**The NDP takes on too many tasks of the GP**	−**3**	−**1**	−**2**	−**2**

−3 indicates that the factor on (weighted) average disagrees most with that statement

3 indicates that the factor on (weighted) average agrees most with that statement

* Distinguishing statements (*p* < 0.01), consensus statements: bold, neutral statements (neglected in final results): italic

*Step 2* Performing Q sorting.

**Fig. 1:**
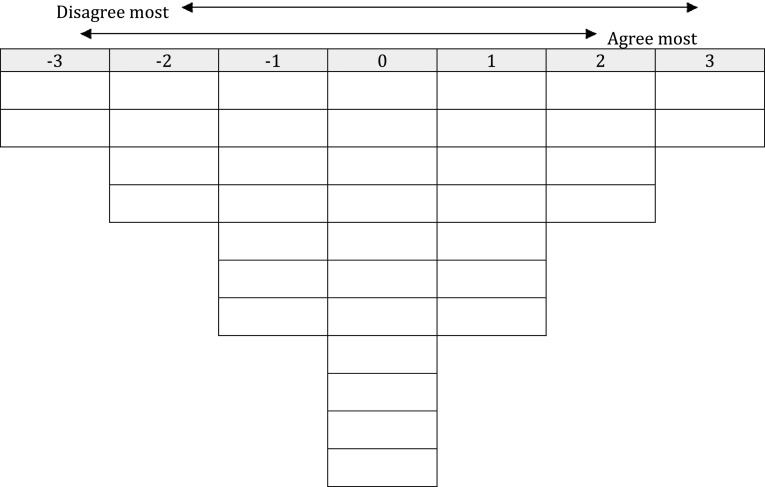
Q sorting table

For Q sorting, respondents considered to have a clear and distinct viewpoint were selected. In this study, they were community, clinical and hospital pharmacists and GPs with varying levels of work experience, located in both rural and urban settings; other pharmaceutical and medical experts; health care insurers; policy makers; practice nurses and patients. Members of the research team approached a convenience sample of respondents for Q sorting online or in person. Q sorting in personal interviews was done by two researchers (AH and AW). Q sorting started by sorting the 37 statements into three categories: ‘agree,’ ‘neutral’ or ‘disagree.’ Next, respondents were asked to place the statements in a Q sorting table (Fig. 1). Respondents were requested to adhere to the Q sorting table, in order to gradually force them to take position on the statements. Q methodology combines statement-sorting and interviews to unravel different perspectives. Therefore, respondents were asked to comment on the four statements at the extreme ends (−3 being disagree most and +3 being agree most). FlashQ© was used as an online Q sorting programme [16].

*Step 3* Analysis of obtained data.

The final step in Q methodology is by-person factor analysis in order to identify significant correlation between individuals, expressed as factors with common viewpoints and preferences [14]. In this study, obtained Q sorts were analysed using PQMethod 2.35 [17]. By-person factor analysis with centroid factor extraction and varimax rotation was conducted with the aim to obtain a clear pattern of relationships between the factors [15]. Since more than the theoretically required minimum of 40–60 respondents was included, it was decided to increase significance [15, 18]. As a result, Q sorts that loaded significantly on one factor (with *p* < 0.01) were included in the analysis. For each factor solution an idealized Q sort was computed. This idealized Q sort represents how a person with a 100 % loading on that factor would have ranked the 37 statements [14, 15].

The content of the factors was examined by reviewing the characterising, distinguishing and consensus statements. Characterising statements are the statements that a factor most (rating +2 or +3) or least (rating −2 or −3) agrees with. The characterising statements are a first peek into the content of a factor. Distinguishing statements are the statements on which factors have different opinions. These statements highlight the differences between factors. Consensus statements are the statements with which all factors (dis)agree. These statements uncover the common viewpoints between factors [14, 15]. Statistical characteristics of the different factor solutions were evaluated.

## Results

A total of 163 participants performed Q sorting: 125 online (77 %) and 38 in person (23 %). Respondents had an average of 17 years of work experience in healthcare (Table 2). Q analysis of the Q sorts supported a maximum of five factors. Content and statistical characteristics were examined for three-, four- and five-factor solutions. The four-factor solution was selected as the desirable solution, based on statistical characteristics, defining statements and written and verbal comments provided by the respondents defining the factors during Q sorting. These four factors explained 53 % of the total variance in the Q sorts (Table 3).

**Table 2:** Baseline characteristics participants

Characteristic	Number
Total number of respondents	163
Female	50 % (n = 82)
Age, mean (range)	45 years (24–77)
Total years of experience in healthcare, mean (range)	17 years (0–42)

Medical and/or pharmaceutical positions	Percentage (n)
Pharmacy	28 % (60)
Community pharmacist	18 % (37)
Non-dispensing pharmacist	4 % (9)
Hospital pharmacist	3 % (7)
Pharmacist trainee	3 % (7)
General practice	16 % (35)
General practitioner	11 % (24)
General practitioner trainee	3 % (7)
Practice nurse	2 % (4)
Other medical and/or pharmaceutical expert	34 % (71)
University teacher or professor	49 % (35)
Medical advisor	17 % (12)
Medical doctor (no GP)	14 % (10)
Researcher	8 % (6)
Employee research and medication safety institute	7 % (5)
Employee health insurance company	4 % (3)
Policy maker	13 % (28)
Pharmacy or medical student	5 % (11)
Patient	2 % (5)

Some participants fulfil multiple positions (e.g. a part-time GP also working part-time as policy maker). As a result 163 participants fulfil 211 positions

**Table 3:** Factor characteristics

Characteristic	Factor			
	A	B	C	D
Number of defining variables	27	50	20	8
Explained variance (%)	15	18	11	9
Cumulative (%)		33	44	53
Correlation between factors				
B	0.59			
C	0.68	0.46		
D	0.68	0.55	0.69	

The next section presents quotations of comments made by respondents (italics). The figures in parentheses, preceded by “s”, correspond to statement numbers in Table 1.

### Similarities between factors

**Fig. 2:**
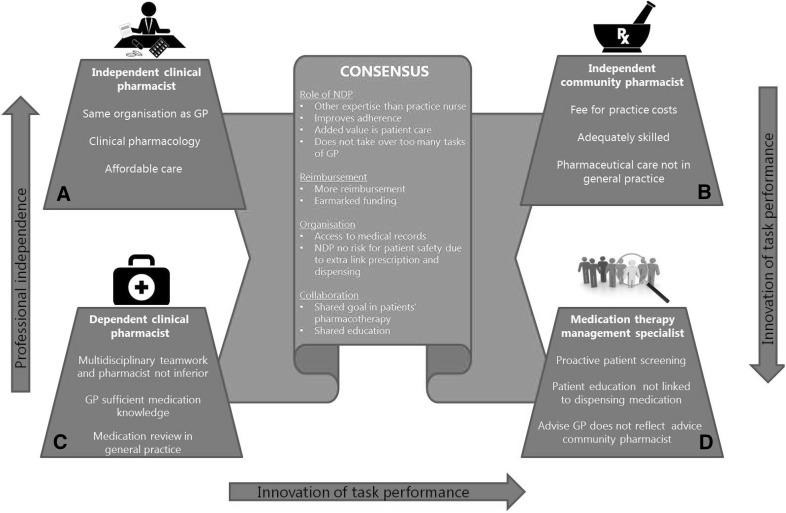
The four factors covering the debate on NDP integration in general practice

All participants shared the same opinion of many statements (Fig. 2).

First, all participants in either factor A, B, C or D believe that an NDP improves adherence (s6), should focus on individual patient care (s18) and does not take over too many tasks of the GP (s37). Second, it is thought evident that the work of an NDP could not be done by a practice nurse (s29). According to some respondents, the pharmaceutical knowledge of the practice nurse is “*nowhere near as extensive as the NDP’s.*” However, some participants suggested that the practice nurse could support the NDPs in the follow-up of some care issues.

Third, all factors emphasize that health insurance companies pay too little for pharmaceutical care (s8) and that there should be funding earmarked for pharmaceutical care (s10): “*Pharmaceutical care is variable and hard to quantify. So it’s challenging for health insurance companies to develop a good reimbursement system.*” This leads to “*low quality patient consultations and medication reviews.*” And “*since reimbursement is insufficient, evaluation and follow*-*up are neglected. Also, quality projects are initiated, but not embedded.*”

Fourth, access to medical records is thought a prerequisite for pharmaceutical care (s20). Numerous participants commented that especially knowledge of (contra-)indications and the results of lab tests are important in providing safe pharmaceutical care. Respondents also stressed the importance of access to medical data: “*Without access to medical records it’s impossible to properly assess the quality of pharmacotherapy and to develop a pharmaceutical care plan tailored to the needs of individual patients.”*

Fifth, another organisational aspect which all factors agree with unanimously is that NDP integration does not pose a risk to patient safety, despite it creating an additional link between prescription and delivery (s3).

Finally, clearly GP and NDP share a common goal in the pharmacotherapy of the patient (s21): “*[Providing good patient care] is indisputable. […] Everything else (costs, practical implementation* etc*.) is secondary*.” Moreover, all respondents agree that pharmaceutical care would be improved by shared training in GP and pharmacist educational programmes (s16).

### Differences between factors

Despite the large number of statements on which all participants shared the same opinion, controversies between the four factors are identified (Fig. 2).

#### Factor A: “independent clinical pharmacist”

Participants aligned with factor A, one-third of whom were medical or pharmaceutical experts (Table 4), seem to fully support NDP integration in general practice. Working in the same organisation is considered necessary to enable successful collaboration between GP and NDP (s28). “*The GP and NDP will share the same vision and principles when they work in one organisation. Integrating an NDP stimulates close collaboration and this will result in unambiguous pharmaceutical care for the patient*.” Since the community pharmacist is not fully informed of the details of the pharmacotherapy of the individual patient (s5), an NDP can provide better pharmaceutical care.

**Table 4:** Defining participants

Expertise	Factor A (n = 27)	Factor B (n = 50)	Factor C (n = 20)	Factor D (n = 8)
	Percentage (n)	Percentage (n)	Percentage (n)	Percentage (n)
Community pharmacist	11 (3)	44 (22)		
Non-dispensing pharmacist	19 (5)	2 (1)		
Hospital pharmacist	7 (2)	2 (1)		25 (n = 2)
Pharmacist trainee		12 (6)		
General practitioner	4 (1)	4 (2)	45 (9)	25 (n = 2)
General practitioner trainee	4 (1)		20 (4)	
Practice nurse	4 (1)		5 (1)	
Other medical and/or pharmaceutical expert	33 (9)	18 (9)	20 (4)	25 (n = 2)
Policy maker	7 (2)	8 (4)		25 (n = 2)
Pharmacy or medical student	11 (3)	10 (5)	5 (1)	
Patient			5 (1)	

Specifically the knowledge about clinical pharmacology that an NDP brings into general practice is regarded as added value (s15). When it comes to complicated patients, the importance of the knowledge of the NDP in primary care is emphasised:

*“The unique combination of an NDP’s knowledge of medication and clinical experience enables him to tailor the pharmacotherapy to the needs of the individual patient. This is particularly important with multimorbidity and polypharmacy, when patients really can’t be treated according to the guideline for one specific disease or condition.”*

Introducing a new care provider in general practice might confuse patients as to whom they should address questions related to medication (s1). Nevertheless, factor A does not identify this as a problem: *“When a clinical pharmacist takes care of a patient, they establish a relationship which makes it natural for the patient to consult them about their pharmaceutical care issues.”* Participants disagree with the statement that an NDP loses their independent position as healthcare provider as an employee of a general practice (s11): “*The clinical pharmacist’s professional integrity will not be influenced by the organisational framework of the workplace.” “An NDP has its own expertise and independency.”* However, participants commented that it will take some time to adjust to this new role of a pharmacist. Respondents loading on this factor disagree with the statement that the NDP takes on too many tasks of the GP (s37). “*The NDP doesn’t take over too much of the pharmaceutical care, but enhances it by working together with the GP*.”

Participants of factor A show confidence in a nationwide introduction of this new pharmacists’ role. This is underlined by the statement that NDP introduction will not make primary care unnecessarily expensive (s12): “*Healthcarecosts might be reduced by preventing adverse effects, overprescribing and medication*-*related hospital admissions*.”

#### Factor B: “independent community pharmacist”

Participants aligned with factor B, over forty percent of whom were community pharmacists (Table 4), insist that the community pharmacist should be the leading independent pharmaceutical care provider, with sufficient financial reimbursement as a prerequisite to perform this role. The participants agree with the statement that a fee for practice costs is necessary to deliver pharmaceutical care (s9): “*Improper reimbursement for pharmaceutical care results in hasty dispensing [pharmaceutical activities performed in a short amount of time] resulting in low quality pharmaceutical care and inadequate follow*-*up.*” The participants aligning with this factor disagree that a community pharmacists is unable to perform pharmaceutical care. This is reflected by their disagreement on: the community pharmacist is insufficiently informed about the patients’ individual pharmacotherapy (s5) and the community pharmacist is not skilled to perform patient consultation (s27). They said, “*patient consultation is the most important part of our job”* and *“during our training, and in the community pharmacy, it’s crucial to have good communication skills otherwise you can’t do your job as a (community) pharmacist.*”

These participants share the opinion that a community pharmacist should advise on the choice of medication (s17): *“Nowadays medication is an important part of therapy. The [up-to-date] pharmaceutical knowledge of a community pharmacist is more extensive than the GP’s knowledge. A community pharmacist can, with this knowledge, increase adherence, efficiency and medication safety by giving advice on the choice of medication.”*

Moreover, these participants disagree with the statement that the patient has more confidence in the NDP than in the community pharmacist (s4). They strongly disagree with the statement that pharmaceutical care can best be accommodated at a general practice (s32) and that clinical medication reviews should take place in the GP practice (s19): “*Medication reviews can also take place in community pharmacy. It’s not really a matter of where the reviews are done, what’s important is that they are done. Medication reviews should be done in collaboration with the prescriber and the patient.*” Therefore, an NDP can also be stationed at a community pharmacy; a potential conflict of interest, due to both consulting on medication and selling it is thought unlikely (s36). Dissimilar to the other factors (A, C and D), participants of factor B strongly support linking dispensing medication and both patient education and giving advice on pharmacotherapy (s31, s26). These statements illustrate the wish to keep general practice and community pharmacy separate. “*Dispensing medication involves more than a GP can handle. GPs have only a limited amount of time per patient. They have little time to give advice on medication, let alone take care of the dispensing*.”

#### Factor C: “dependent clinical pharmacist”

The theme of this factor is pharmaceutical care improvement, managed primarily by GPs, with a supporting role for the NDP to join the team as a dependent pharmaceutical care provider. Sixty-five percent of the participants defining this factor were GPs or GP trainees (Table 4).

Unlike the other factors (A, B and D), participants in factor C believe that the GP has enough knowledge of medication (s13): “*In general, no major accidents happen due to the GP’s pharmacotherapeutic choices. Over the past years, the GP’s knowledge has increased.*” However, GPs are considered to be open to having more healthcare providers in their practice and encourage multidisciplinary teamwork (s2): “*The support from other caregivers is very nice, since a doctor can’t know it all*.” In line with this multidisciplinary approach, participants aligning with this factor are open to having an NDP in their practice and debate the statement that a pharmacist has an inferior position which could impede medication safety (s23): “*A pharmacist is not inferior. Collaborating on conducting safe practice together is the main issue.*” Medication safety is not thought endangered by inequality in positions but “*a lack of collaboration or organisational flaws*” are considered the most likely cause of medication safety problems.

Those aligning with this factor are the only respondents who agree with the statement that an NDP cannot be employed at a community pharmacy due to a conflict of interest (s36). They agree with the statement that the patient has more confidence in the NDP than in the community pharmacist (s4): “*Patients associate general practice with good quality of care. They prefer to discuss their care issues with healthcare providers who are physically present in general practice.*” Therefore, clinical medication reviews should be organised in general practice (s19): “*The GP is the centre point of primary care. That’s why it’s logical to do medication reviews in general practice*” and “*the access to medical records in general practice facilitates medication reviews.*” Pharmaceutical care provision can be performed in close collaboration with an NDP but in contrast to the respondents aligning with factors A and B, respondents on factor C disagree with the statement that an NDP should be an independent prescriber (s34): “*A pharmacist is not a medical doctor.*”

#### Factor D: “medication therapy management specialiste-”

Factor D supports the idea of integrating an NDP in primary care and shows similarities with factors A and C, although this vision of the added value of an NDP includes managerial expertise. Also, this factor shows some mistrust in the ability of community pharmacists to provide good pharmaceutical care. Participants defining this factor are a heterogeneous group of GPs, hospital pharmacists, policy makers and other medical and/or pharmaceutical experts (Table 4).

The added value of an NDP is made tangible by their proactive task to screen patients with potential drug therapy problems (s25): “*An NDP can intervene before medicines are prescribed, while intervening afterwards is inconvenient, time*-*consuming and confuses the patient.*” A GP who worked with an NDP stated: “*In our practice, the NDP’s particular expertise to proactively identify high risk patients resulted in improved patient safety.*” Besides this preventive approach, factor D is most outspoken about the individual patient care an NDP should deliver (s18). Also, they are most distinct about the NDP not being an independent prescriber (s34): “*Prescribing and monitoring medication have to be separate at all times*” and “*the GP will lose control over patient care if multiple healthcare professionals are allowed to prescribe medication independently” and “the pharmacist has not enough (clinical) knowledge about a patient.*” Despite the latter, the GP conceded their insufficient knowledge of medication (s13): “*GPs get very little schooling on medication.*”

This factor suggests that educating patients on their pharmacotherapy can be separated from dispensing medication (s31). Moreover, it was stated that the information on medication given by the community pharmacist to the patient does not reflect the GP’s advice well enough (s22): “*Unfortunately, patients are often confused by the different advice in the community pharmacy.*” Also, participants aligning with this factor disagree on the statement that a fee for practice costs for community pharmacists is essential to enable delivery of pharmaceutical care (s9). This implies that this factor does not necessarily support the development of community pharmacists as pharmaceutical care providers. On the other hand, participants aligning with factor D acknowledge that a community pharmacists’ primary concern is not the financial status of the pharmacy business (s7), which suggests the possibility of another primary concern, for instance pharmaceutical care.

## Discussion

We systematically mapped the debate amongst stakeholders on introducing an NDP in primary care practice and revealed four perspectives: “the independent community pharmacist” (Factor B), “the independent clinical pharmacist” (Factor A), “the dependent clinical pharmacist” (Factor C) and “the medication therapy management specialist” (Factor D).

Factors A, C and D favour NDP integration in primary care practice. The main contrast between factors A and C concerns the level of professional independence, which is an eminent point of debate when introducing new roles into current practice. Fournier says that the construction of boundaries and the creation of an independent area of knowledge is crucial to professional development [19]. In accordance with this, factor A supports the integration of an NDP as an “independent clinical pharmacist” based upon the clinical knowledge that an NDP brings into practice and the benefits of working within the same organisation. This creates interprofessional trusting relationships and integrates work processes, thereby improving quality and continuity of individual patient care. Despite the restricted clinical, economic and political autonomy of pharmacists described by Edmunds [20], factor A highlights development in the process of reprofessionalisation of pharmacy.

In contrast to factor A, factor C stresses the role of an NDP in general practice as a “dependent clinical pharmacist” within a multidisciplinary team of healthcare professionals, with drug monitoring and not drug prescription as the primary task. This perspective accentuates the GPs’ wish to maintain professional dominance, triggered by external threats of their privileged position [8]. Also, it is acknowledged that GPs are hesitant about the clinical roles of medication management performed by community pharmacists [2]. This hesitance towards community pharmacists might have influenced their perception of the level of independence that an NDP in primary care practice should attain.

Factor D is distinct about the innovation level of tasks performed by NDPs. Supporters of this factor promote a new model of care: an NDP as a “medication therapy management specialist” who focuses on proactive screening (and treating) of patients with potential drug therapy problems, thereby integrating managerial expertise and values into the professional work. It involves population-focused preventive care, which is important in an era with a large ageing population, to prevent avoidable chronic diseases and unnecessary medical expense [21].

While factors A, C and D favour NDP integration in primary care practice, factor B see pharmaceutical care provision improved by maintaining and expanding the traditional roles of community pharmacists. The respondents aligning with factor B underline the essential role of community pharmacists as leading pharmaceutical care providers [22]. According to the respondents of factor B, pharmaceutical care, including dispensing medication, should definitely not be accommodated in general practice. Factor B wishes to enhance the level of independence of community pharmacists in the context of treating individual patients to legitimate their role as pharmaceutical care professionals [20]. They see clear boundaries and the creation of an independent area of knowledge crucial to the professional development of the pharmacist [19].

Although these four perspectives are distinct, we identified a relatively large overlap between them. There was consensus on the potential of the NDP as a pharmaceutical care provider. Moreover, all respondents in this Q study were consistent in their view on financial, organisational and collaborative issues such as more funding for pharmaceutical care improvements, better access to medical records for pharmacists, shared education for GPs and pharmacists, and shared responsibility for the outcome of pharmacotherapy. This high level of consensus demonstrates a willingness for interprofessional collaboration and a positive attitude towards different aspects of an NDP integrated in primary care practice.

A strength of this study is that it included participants with a large variety of medical and pharmaceutical experience. This makes it likely that it represents all the different viewpoints on the NDP in Dutch general practice. No indications for missing topics were found in the evaluation by the expert group and pilot study. Also, this study is part of the POINT study, a large multicenter intervention study on NDPs in Dutch primary care practice [11] and the results of this Q study will contribute to further development of the intervention.

This study does have limitations. Firstly, nothing can be said about the prevalence of the four factors amongst pharmacists, GPs and external stakeholders in the wider population since Q methodology is not designed for this purpose. Secondly, for pragmatic reasons the majority of the respondents ranked the Q set electronically, including computer-based interviews instead of personal interviews. In-person interviews enable the researcher to better understand and interpret the results. However, we identified no apparent differences in reliability or validity of these two methods of administration [23].

Since all stakeholders underline the potential benefit of an NDP as pharmaceutical care provider, we need to reflect upon the financial aspects of these services. As said, all stakeholders agree that more and earmarked funding is needed to improve pharmaceutical care. In the POINT study that we are currently evaluating, the NDP services were funded via a temporary grant [11]. A sustainable model of reimbursement for the services performed by NDPs is needed. The employer could than either be community pharmacies or GP practices. A community pharmacy fee finance model, however, is less feasible because this model is based on dispensing of medication. The relatively small fees for pharmaceutical services obstruct employment of an NDP in community pharmacies. Implementation through the GP fee finance model is feasible, but limited to groups of collaborating GP practices. Implementation of the NDP would probably be optimal if dedicated additional funding from the insurance company. Whether and how an NDP can be employed in other health care systems heavily depends on the local situation. Hence, it would be relevant to replicate this study in a country with a different health care system.

It is important to define the scope of practice of NDPs in comparison to both the community and clinical pharmacists. The NDP is the clinical pharmacist in primary care. While earlier initiatives to bring hospital clinical pharmacists in primary care failed, the NDP provides an alternative role. NDP services will add especially to the quality of pharmaceutical care of specific subgroups of individual patients, such as elderly patients and those with polypharmacy. The community pharmacist will—in addition to dispensing medication and medication surveillance—provide pharmaceutical care connected to the pharmaceutical product to less complex patients. In contrast to the UK and the US, neither community pharmacists, clinical pharmacists nor NDPs can prescribe drugs in the Netherlands. In the current study prescribing by pharmacists was not seen a priority for an NDP.

## Conclusion

Despite the fact that introducing new professional roles in healthcare can be controversial, this Q study identified a consensus on various features of the NDP role, as well as on financial, organisational and collaborative aspects of NDP integration in primary care practices. This shows the potential of an NDP as a pharmaceutical care provider and the willingness for interprofessional collaboration. The main identified controversies concern the NDP’s level of professional independence and the level of innovation of task performance. The results from the POINT intervention study will be an important next step in resolving current controversies.
